# Supplementary figures and images for: Portable and accurate diagnostics for COVID-19: Combined use of the miniPCR thermocycler and a well-plate reader for SARS-CoV-2 virus detection
Source: PLoS One. 2020 Aug 13;15(8):e0237418. doi: 10.1371/journal.pone.0237418 (PMC7425953; doi:10.1371/journal.pone.0237418)

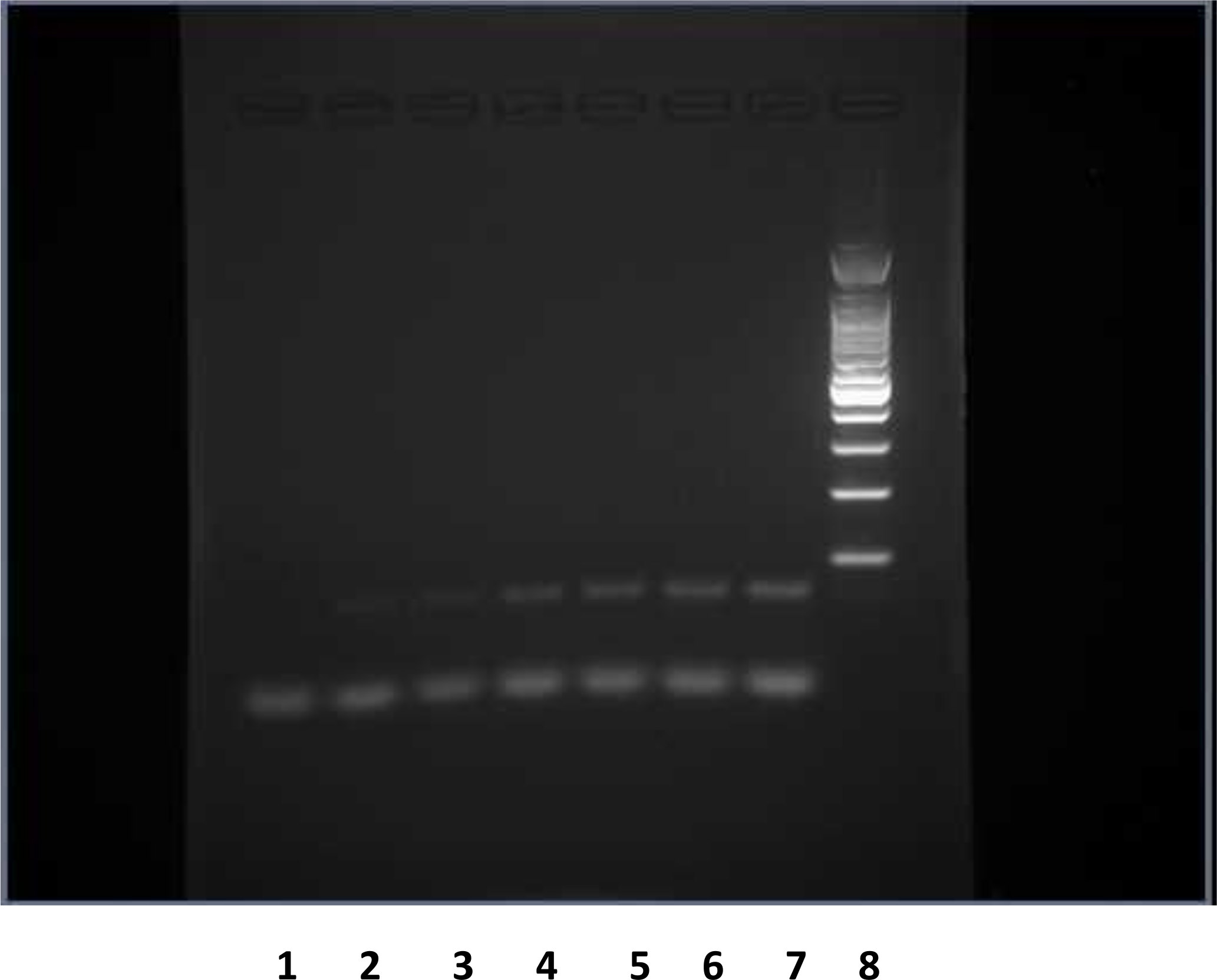

Supplement: S1 Fig — PCR was performed using a miniPCR® thermocycler. The primer set N1 was used. The initial template amount was gradually increased from left to right: negative control (lane 1), 625 copies (lane 2), 2.5 × 10^3^ (lane 3), 1.0 × 10^4^ (lane 4), repetition of 1.0 × 10^4^ (lane 5), 4.0 × 10^4^ (lane 6), 2.0 × 10^5^ DNA copies (lane 7), and molecular weight ladder (lane 8). (TIFF) [file pone.0237418.s002.tiff]

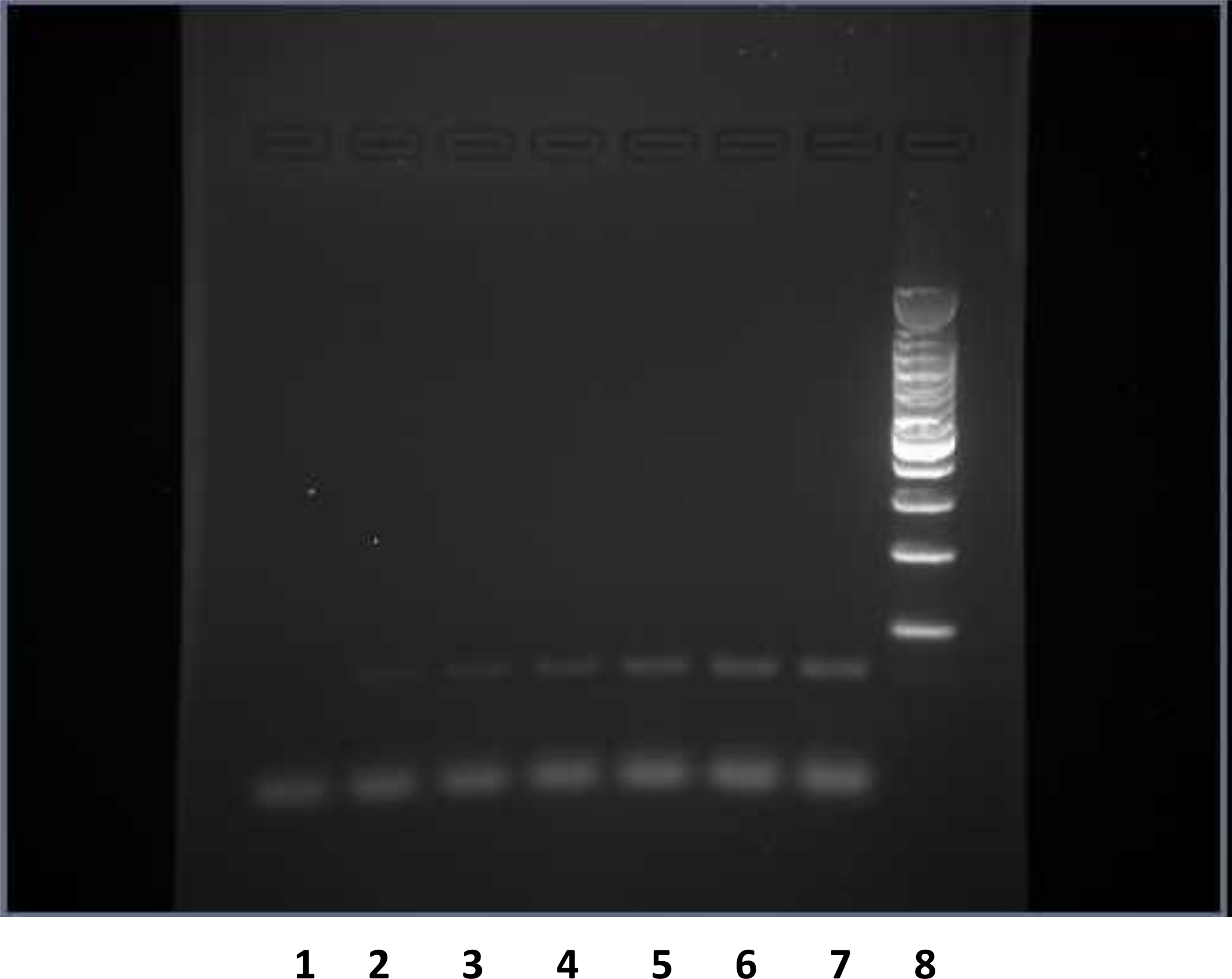

Supplement: S2 Fig — PCR was performed using a miniPCR® thermocycler. The primer set N2 was used. The initial template amount was gradually increased from left to right: negative control (lane 1), 625 copies (lane 2), 2.5 × 10^3^ (lane 3), 1.0 × 10^4^ (lane 4), repetition of 1.0 × 10^4^ (lane 5), 4.0 × 10^4^ (lane 6), 2.0 × 10^5^ DNA copies (lane 7), and molecular weight ladder (lane 8). (TIFF) [file pone.0237418.s003.tiff]

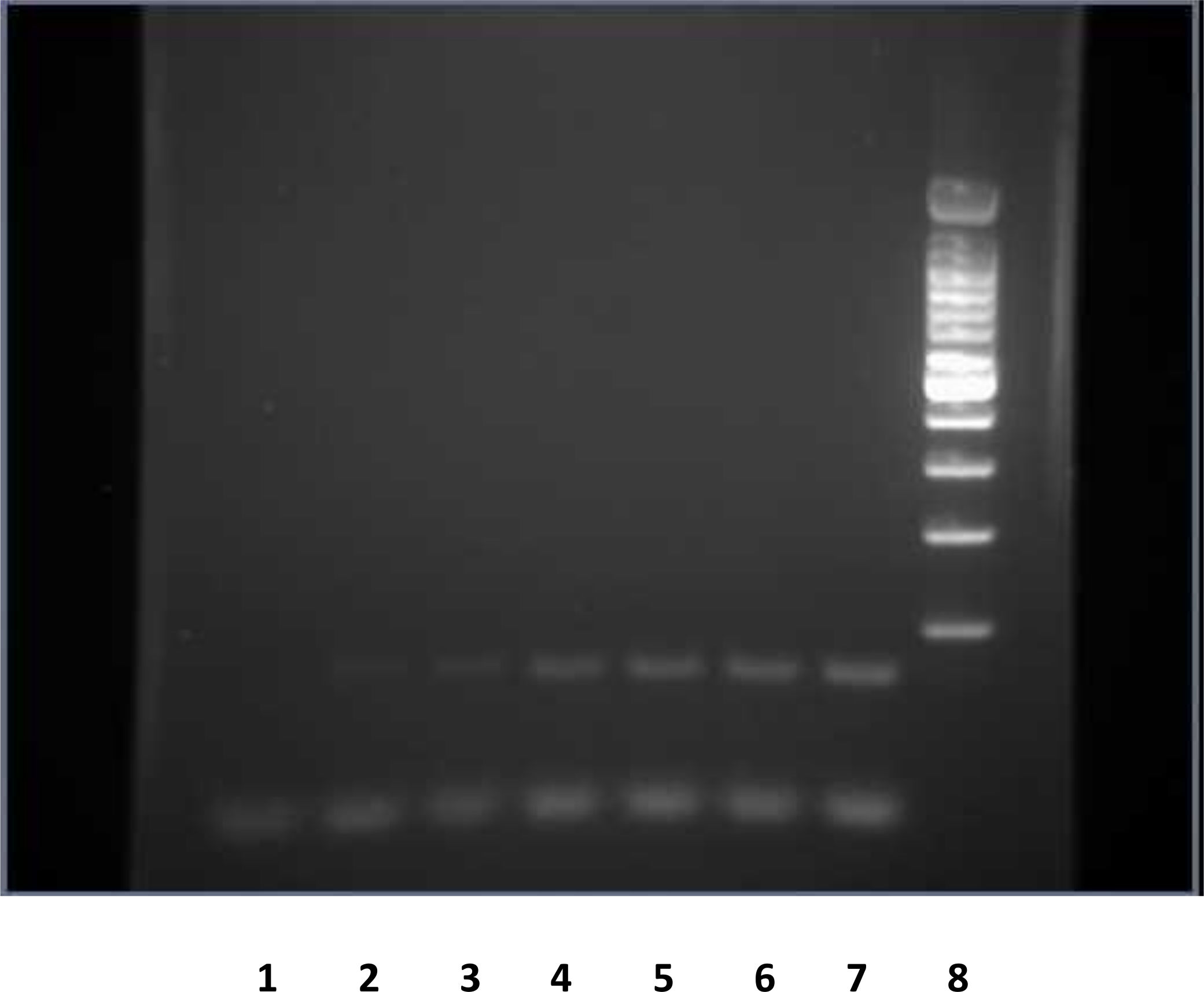

Supplement: S3 Fig — PCR was performed using a miniPCR® thermocycler. The primer set N3 was used. The initial template amount was gradually increased from left to right: negative control (lane 1), 625 copies (lane 2), 2.5 × 10^3^ (lane 3), 1.0 × 10^4^ (lane 4), repetition of 1.0 × 10^4^ (lane 5), 4.0 × 10^4^ (lane 6), 2.0 × 10^5^ DNA copies (lane 7), and molecular weight ladder (lane 8). (TIFF) [file pone.0237418.s004.tiff]
